# Supplementary figures and images for: A divergent cyclin/cyclin-dependent kinase complex controls the atypical replication of a malaria parasite during gametogony and transmission
Source: eLife. 2020 Jun 22;9:e56474. doi: 10.7554/eLife.56474 (PMC7308089; doi:10.7554/eLife.56474)

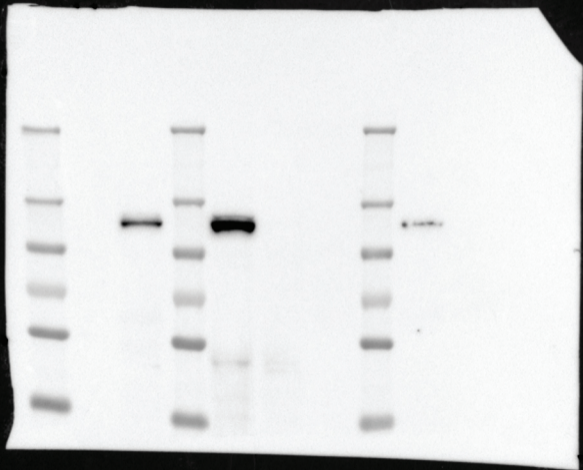

Supplement: Figure 1—source data 2. [file elife-56474-fig1-data2.pdf]

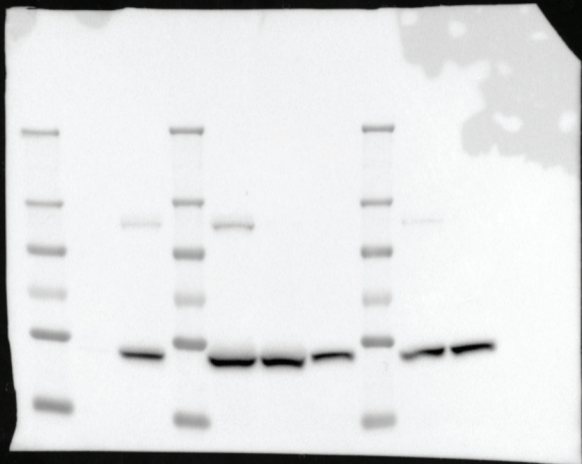

Supplement: Figure 1—source data 3. [file elife-56474-fig1-data3.pdf]

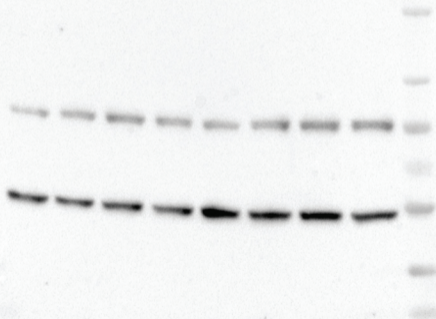

Supplement: Figure 4—source data 1. [file elife-56474-fig4-data1.pdf]

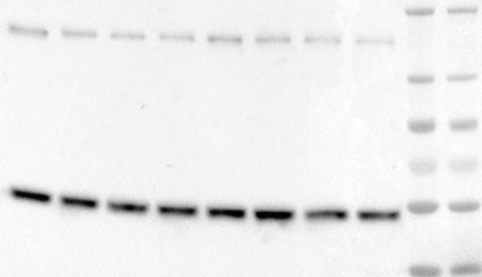

Supplement: Figure 4—source data 2. [file elife-56474-fig4-data2.pdf]
